# The Association between Varicella Vaccination and Herpes Zoster in Children: A Semi-National Retrospective Study

**DOI:** 10.3390/jcm12134294

**Published:** 2023-06-27

**Authors:** Ester Forer, Adi Yariv, Daniel Ostrovsky, Amir Horev

**Affiliations:** 1 Pediatric Division, Soroka University Medical Center, Beer-Sheva 8410101, Israel; esti.forer@gmail.com; 2 Clinical Research Center, Soroka University Medical Center, Ben-Gurion University of the Negev, Beer-Sheva 8410101, Israel; yarivad@post.bgu.ac.il (A.Y.); ostrdani@post.bgu.ac.il (D.O.); 3 Faculty of Health Sciences, Ben-Gurion University of the Negev, Beer-Sheva 8410501, Israel; 4 Pediatric Dermatology Service, Soroka University Medical Center, Yitzhak Rager Ave., P.O. Box 151, Beer-Sheva 8410101, Israel

**Keywords:** varicella zoster, herpes zoster, vaccination

## Abstract

Varicella vaccination in children has been performed worldwide in recent years. Despite established effectiveness, many countries still do not routinely vaccinate children against varicella, probably due to concerns about complications, such as herpes zoster infection. We aimed to compare the herpes zoster incidence in children before and after implementing the mandatory varicella vaccine in Israel in 2008. As a secondary aim, we characterized several parameters, including age, sex, and ethnic sector among herpes zoster cases, and we evaluated the complication rate to identify data relevant to the immunization status of the pediatric population. A retrospective study was conducted between 2000 and 2021, including patients aged 0–18 years old in a large cohort in southern Israel. A time series analysis and complication rates evaluations were performed in the pre- and post-vaccination eras. A total of 109.24 herpes zoster cases per 100,000 population per year were diagnosed between 2000 and 2007 (pre-vaccination era), compared to 354.71 herpes zoster cases per 100,000 population per year diagnosed between 2008 and 2021 (post-vaccination era) (*p* < 0.001). No change in the complication rate was documented. Thus, we concluded that there is an association between the varicella vaccine implementation program and the increase in the rate of herpes-zoster occurrence without a concurrent negative contribution to herpes zoster-related morbidity.

## 1. Introduction

Varicella zoster virus (VZV) is a virus of the family *Herpesviridae*. VZV affects only humans, and the clinical manifestations of VZV infection are classified as varicella (chickenpox) or herpes zoster (HZ) (shingles). The virus primarily infects children, manifesting as chickenpox, and it is characterized by the cutaneous distribution of diffuse and scattered maculopapular vesicles. VZV then becomes latent in the nervous system. As cellular immunity to the virus diminishes with age or in persons in an immunocompromised state, reactivation of the latent VZV may occur, possibly resulting in shingles, which is characterized by unilateral vesicular eruptions within the affected dermatomes (but other presentations, such as zoster sine herpete, in which skin itches and/or hurts without a rash, can also occur). This condition could potentially cause shingles-associated complications, including postherpetic neuralgia, ocular involvement, and central nervous system disease [1].

Varicella is considered a self-limiting disease that primarily infects children. In 2014, the World Health Organization (WHO) estimated approximately 4.2 million varicella cases with severe complications and around 4200 related deaths per year worldwide [2]. In France, the disease was associated with severe complications in 3% of patients younger than 15 and 6% of patients older than 15, including secondary bacterial infections of the skin and lungs, sepsis, aseptic meningitis, encephalitis, and Reye syndrome [3].

The United States became the first country to implement a routine childhood varicella vaccination program after the vaccine was licensed in 1995 [4]. The Centers for Disease Control (CDC) recommends two doses of the varicella vaccine for children, adolescents, and adults who have never had chickenpox and have never been vaccinated. Children are routinely recommended to receive the first dose at 12 to 15 months of age and the second dose at 4 to 6 years of age [5]. In Israel, a mandatory varicella vaccination program was established in 2008, requiring two doses at 1 and 6 years of age.

In December 2014, varicella vaccines were recommended in 33 predominantly higher socioeconomic countries where the vaccination program was not fully implemented, implying that, despite established effectiveness, many countries still do not routinely vaccinate children against varicella [6]. The reasons for the low adoption rate could include implementation costs or an increased age of onset, which is associated with a clinically severe presentation.

Additional possible reasons for low vaccine adoption rates involve concern about complications. These complications may be divided into those originating from the vaccine strain varicella virus or the later complications. Immediate post-vaccination complications may include life-threatening infections caused by the vaccine strain, such as pneumonitis with a generalized varicelliform rash [7] and postherpetic neuralgia and meningoencephalitis with or without rash [8], which are much more common in immunodeficient populations, while the increase in the incidence of HZ is a relatively late complication [9] that may impose additional complications, such as severe ophthalmic and dermatological involvement, including HZ keratoconjunctivitis and eyelid dermatitis, which may affect both immunodeficient [10] and healthy [11] populations.

Several models of varicella vaccination impact have studied the hypothesis stating that contact with varicella cases causes exogenous boosting of specific immunity to zoster [12,13,14,15,16,17]. These models have suggested that routine infant varicella vaccination will cause zoster incidence to increase in the medium term. However, in the medium to long term (30–75 years after vaccination), zoster incidence will decrease to levels less than what they were prior to vaccination. Based on these data, a European union guidance document stated that higher coverage, greater vaccine efficacy, and two-dose vaccination programs are predicted to produce the greatest medium-term increases but to decrease zoster incidence in the long term [18].

Our study addressed this specific vaccine-related phenomenon, which could generate controversy. To shed light on whether or not the vaccine against varicella contributed positively to HZ-related morbidity and complications, the primary aim of the present study was to compare HZ incidence before and after the mandatory varicella vaccination program was implemented in a large cohort in southern Israel. In addition, we conducted a stratified analysis for age, sex, and ethnic sector. Finally, we further evaluated the HZ complications rate. Thus, we were able to infer data that might be relevant to the pediatric population’s HZ morbidity in light of its immunization status.

## 2. Materials and Methods

This retrospective ecological study was conducted between 2000 and 2021. In 2008, the beginning of the mandatory vaccination of children was conducted by the state of Israel. Therefore, we conducted an interrupted time series analysis to assess the association between the varicella vaccination and HZ cases.

Subjects diagnosed with HZ identified based on International Classification of Disease (ICD)-9 codes or having a positive lab result for HZ (PCR, biopsy, Tzanck smear, or serology results) between 2000 and 2021, aged 0–18 years old, were included in the HZ-exposed group. Diagnoses were performed by the patients’ primary physicians, dermatologists, or neurologists. Thus, we believe that a relatively small number of zoster sine herpete cases (skin itches and/or hurts without a rash) were included.

In addition, our study evaluated complication rates by collecting relevant ICD-9 codes and comparing them in the pre- and post-vaccination eras.

The study population comprised of patients insured within the southern district of Clalit Health Services (CHS) or seen at Soroka University Medical Center (SUMC). CHS is the largest public healthcare provider organization in Israel and serves more than half of Israel’s population, covering approximately 4,600,000 people (and around 1,000,000 people in southern Israel). SUMC, with 1200 beds, is the only tertiary hospital in the south of Israel, covering a massive geographical area. 

Data were extracted from a digital system called “Bina” that encompasses information on all children aged 0–18 years old who live in Israel’s southern district and are insured by the CHS. Subject confidentiality was maintained throughout the study. A unique subject identification code was used to identify all data reported for each subject to ensure that the data could be traced back to the source data. Data relating to the study might be made available to third parties (e.g., in the case of an audit performed by regulatory authorities) provided that the data are treated confidentially, and the subjects’ privacy is guaranteed.

Categorical variables are presented as counts and percentages of the total. Population size was determined at the mid-point time interval and used as the denominator to estimate the incidence. We used the chi-square test to compare incidence between time intervals. The analysis was conducted using R software version 4.2.2, and the results were considered statistically significant at a *p*-value of 5%.

## 3. Results

**Figure 1:**
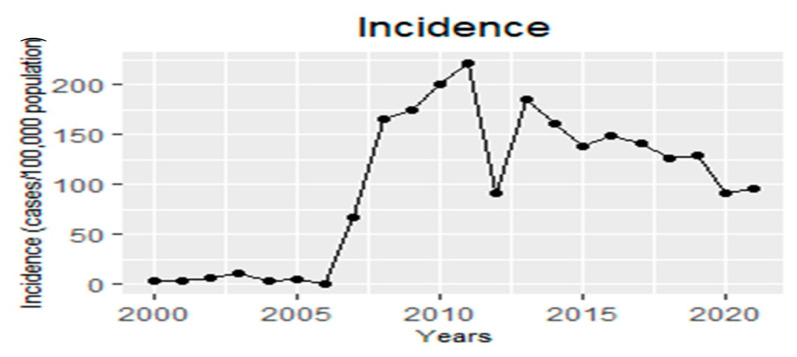
General herpes zoster incidence (cases/population) × 100,000 per year.

A total of 2895 HZ cases were included in the research period. The data regarding the study population, including the children’s age groups upon diagnosis with HZ, sex, and ethnic sector, are presented in Table 1:

Between 2000 and 2007, 109.24 HZ cases per 100,000 population per year were documented. However, a significant increase in the incidence rate of infected children was observed from 2008 to 2021, with a total of 354.71 HZ cases per 100,000 population per year (*p* < 0.001). Figure 1 demonstrates the HZ incidence rate before and after the initiation of mandatory varicella vaccination in 2008.

### 3.1. Age

**Figure 2:**
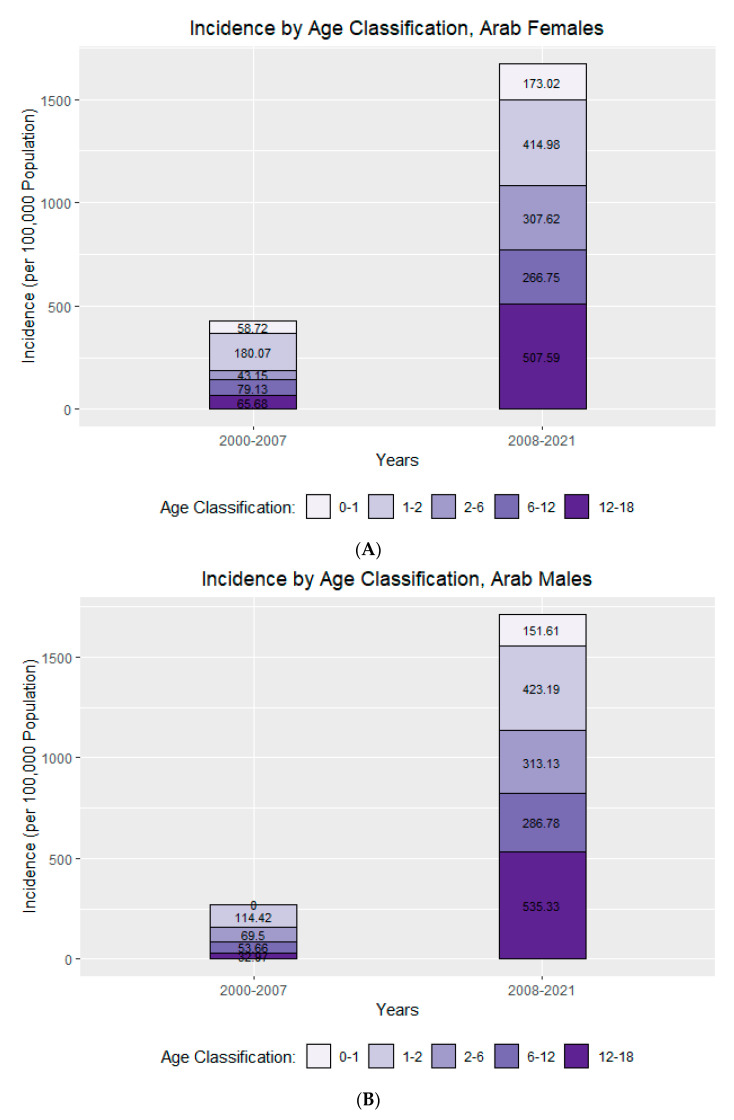

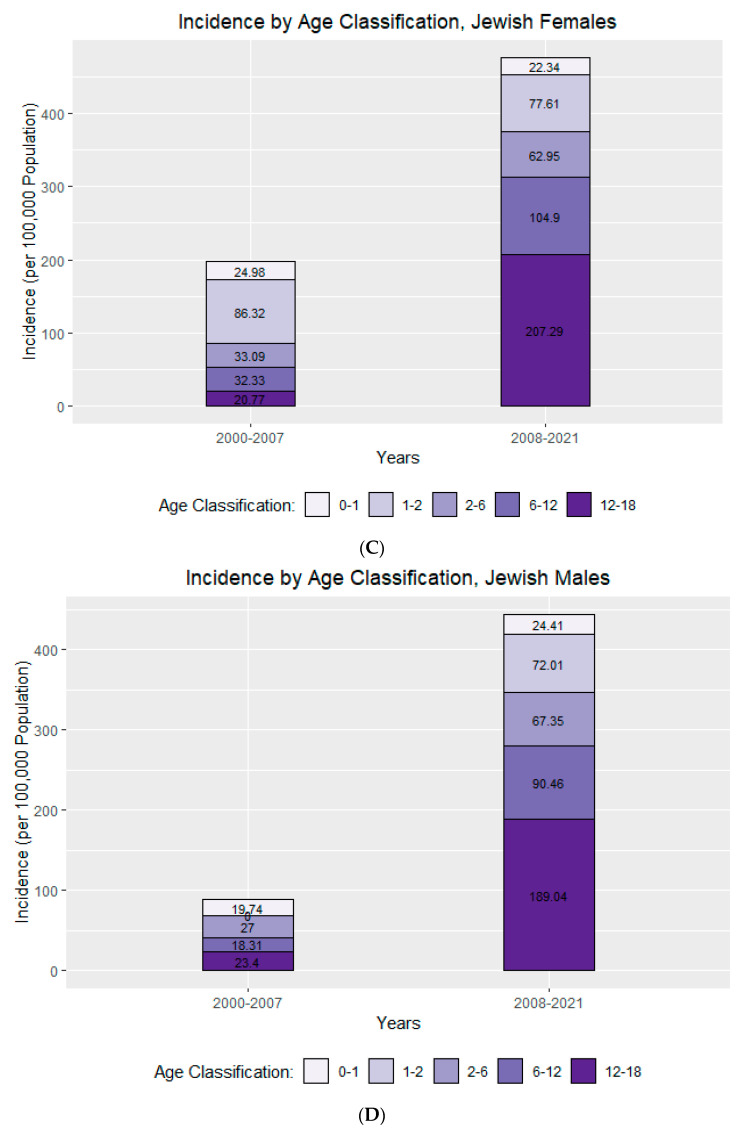
(**A**) Herpes zoster incidence by age, Arab females [cases (by age)/population (by age)] × 100,000 per year. (**B**) Herpes zoster incidence by age, Arab males [cases (by age)/population (by age)] × 100,000 per year. (**C**) Herpes zoster incidence by age, Jewish females [cases (by age)/population (by age)] × 100,000 per year. (**D**) Herpes zoster incidence by age, Jewish males [cases (by age)/population (by age)] × 100,000 per year.

The results showed that, in most age groups, a distinct increase in HZ cases occurred in the post-vaccination era. The most significant increase occurred in adolescents aged 12–18 years old, with an average increase of 19.71% in HZ cases per 100,000 population in the post-vaccination period compared with the pre-vaccination period (Figure 2A–D).

### 3.2. Gender

**Figure 3:**
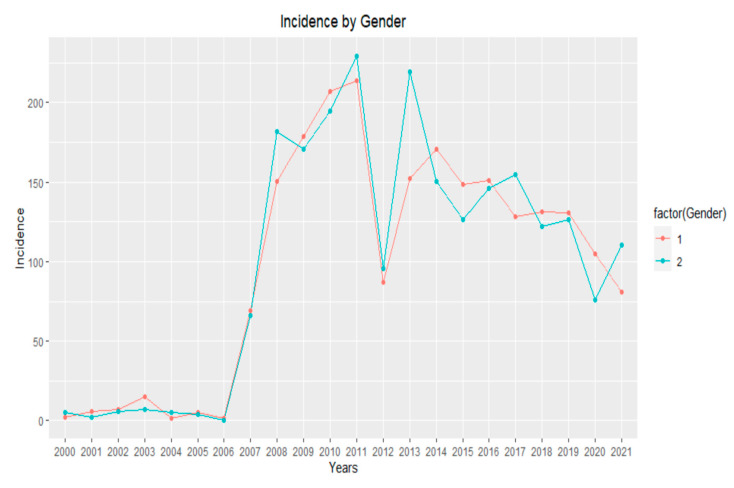
Herpes zoster incidence by gender [cases (by sector)/population (by sector)] × 100,000 per year.

An additional classification according to gender groups was conducted for the pre- and post-vaccination periods. Female subjects were more affected than male subjects between 2000 and 2007, with an average of 78.03 HZ cases per 100,000 population per year versus 44.87 HZ cases per 100,000 population per year among male subjects. In contrast, both female and male subjects were almost identically affected between 2008 and 2021, with an average of 238.33 HZ cases per 100,000 population per year among female subjects versus an average of 239.244 HZ cases per 100,000 population per year among male subjects (Figure 3).

### 3.3. Ethnicity

**Figure 4:**
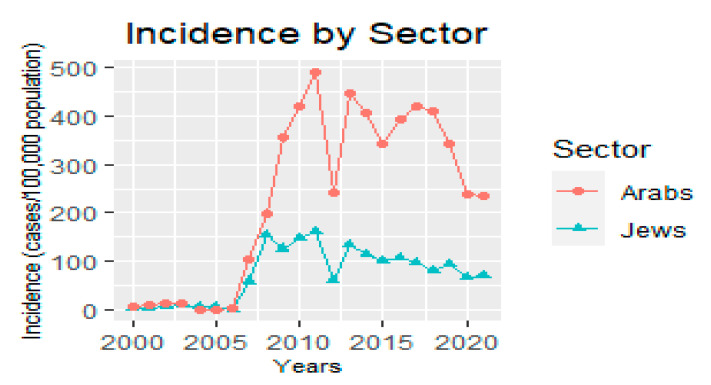
Herpes zoster incidence by ethnic sector [cases (by sector)/population (by sector)] × 100,000 per year.

Another subdivision of HZ cases was created according to ethnic sector (Jews and Arabs). We found that, until 2007, the number of HZ cases was similar in the two ethnic sectors (an average rate of 10.1 cases per 100,000 population for the Jewish sector and 26.98 cases per 100,000 population for the Arab sector). However, from 2008, a significant increase in the number of HZ cases was observed in the Arab sector, while a less dominant increase was observed in the Jewish sector (an average rate of 96.56 cases per 100,000 population for the Jewish sector and 255.24 cases per 100,000 population for the Arab sector; Figure 4).

Seventy-eight HZ cases were documented in 2007, with sex and age characteristics similar to those found in the post-vaccination era (female-to-male ratio, 0.9; mean age, 8.9 ± 4.9 years old, resembling the sex and age distributions of the patients in the post-vaccination period at 0.97 and 8.9 ± 5.2, respectively). In addition, chi-square testing revealed a significant increase in HZ cases between 2007 and 2008 (*p*-value < 0.001).

Upon comparing both sectors and several age groups, we found that the increase in HZ incidence per 100,000 population among children (both male and female) was greater in the Arab sector. The growth in the Arab female group was significantly higher compared to other groups of all ages (an average incident rate increase of 41.05 times for the Arab female subjects, 7.45 for the Arab male subjects, 3.38 for the Jewish female subjects, and 4.19 for the Jewish males subjects).

A decrease was observed only in Jewish girls aged 0–2 years old (11% decrease in 0–1, 10% decrease in 1–2). However, in other age ranges within this group, there was an increase in the number of cases, albeit a significantly moderate increase compared to other groups, as shown in Table 1.

### 3.4. Herpes-Zoster Complication

No complications involving the central nervous system were found in our study population. Additionally, no deaths or major disabilities were found among our study populations. On the other hand, ophthalmic and dermatological complications were found in the two periods, including HZ keratoconjunctivitis and eyelid dermatitis. Due to the relatively small number of complications and in accordance with the mandatory varicella vaccination program implemented in Israel in 2008, we compared the cumulative incidence rates between 2000–2007 and 2008–2021. Our results showed no significant change in the incidence of complications before and after 2008 (0.25 versus 12.07 complications per 100,000 population, *p*-value = 0.9976; Table 2).

## 4. Discussion

The current study documented a significant increase in the HZ incidence rate after the varicella vaccine became mandatory in Israel in 2008. The changes in the number of HZ cases in Israel between 2000 and 2021 may be strongly related to the vaccination program undertaken by the government and thus could contribute to the discussion regarding the controversy of whether the vaccine has an impact on HZ-related morbidity.

Before the introduction of routine varicella vaccination, the reported HZ incidence rates among children and adolescents worldwide ranged from 42 to 220 per 100,000 person-years [19,20]. Experts’ opinions over the past few years have led to research concluding that HZ frequency and severity are less after varicella vaccination than after wild-type varicella infection [21,22]. For example, during a period with high varicella vaccine coverage (2003–2014), a 72% decrease in overall HZ incidence in children < 18 years of age was reported in a study conducted in the United States, which included a population totaling 199,797 children from multiple health centers [23]. A similar decline in HZ rates after 2006 in children 0–17 years old was demonstrated in the United States by Harpaz et al. [20]. The formal CDC vaccine information statement also supports this observation [24]. On the other hand, the annual incidence of HZ did not change significantly in children in Turkey, according to a large study investigating 1,090,803 patients, both children and adults, with a time trend analysis of 9 years [9]. Another study conducted in Japan expected HZ morbidity to increase among younger individuals in Japan in the upcoming years [25].

Despite the decline mentioned above in the United States and arrest in Turkey in pediatric HZ cases following varicella vaccination, there have still been reports regarding post-vaccination HZ cases. Most have been found in immune-deficient populations, while some have occurred in immunocompetent children, either in the same dermatome in which the vaccine was injected or at a remote location. In some cases, the zoster rash can be as severe as that following wild-type varicella [9,11,25].

To address whether varicella vaccination contributed positively [20,24] or negatively [9,11] to HZ-related morbidity, we consider four notable findings in our study. First, we consider the year 2007 to be an indicator. We assume that the increase in HZ cases with similar age, sex, and sector characteristics to those of the post-vaccination era, when vaccinations were not yet mandatory but began to be offered to specific populations, suggests a possible correlation between varicella vaccination and HZ occurrence. However, precise data on the use of vaccines before vaccination became mandatory were unavailable; thus, direct causality could not be determine. We also cannot account for the youngest age group. The relatively short latency period between the time of vaccination and HZ appearance could be explained by the high immunogenicity that a live attenuated vaccine has. Former studies might not have reported similar phenomena due to unknown factors (for instance, fewer post-vaccination clinic/hospital visits resulting from proper guidance before receiving the vaccine, such as that given in the CDC vaccine information statement, greater awareness of possible immediate and late-onset rash eruptions following injection, different health system structures, etc.). Additional explanations for the punctuative increase in HZ cases during 2007 might be related to other non-vaccine-related factors, but they require further study. Second, we used the unique characteristics of our local populations, specifically in the Arab sector. The Arab population mainly lives in separate villages and towns in the southern Israel district. The population data were taken from community data, in which the sector is also defined by settlement. Thus, a direct association could be determined between the number of HZ cases and the two distinct ethnic sectors. Additionally, the Arab population in the southern Israel district has previously been proven to adhere more to routine vaccination programs involving school-aged children [26], which may imply that adherence within other age groups is greater among Arabs than among Jews. In light of this possibility, the more significant increase in HZ cases in the Arab sector post-vaccination strengthens the possible causal association between varicella vaccination and HZ occurrence. Third, the change in the number of HZ cases was predominantly observed in the older age group (children aged 12–18 years old) in the post-vaccination era, rather than in the pre-vaccination period. This finding is in agreement with the physiology of the disease [24] since reactivation of the latent VZV is preceded by several years of virus latency in the dorsal root ganglia. It is expected that, if vaccination does affect disease rates, it will be most significant in the older age group due to its longer time interval since vaccination, which allows reactivation to occur, as indicated in our study. Finally, as was biologically proven in the past, wild-type virus strains are associated with a greater risk of complications [19,27]. Therefore, an increase in HZ complications per 100,000 individuals is expected without vaccination. This statement aligns with our finding of similar HZ dermatological and ophthalmic complications incidences and HZ complications incidences per 100,000 population in the pre-vaccination period.

Thus, we assume that Israel’s mandatory varicella vaccination program contributed positively to avoiding severe HZ morbidity and related complications.

### 4.1. Strengths

Our research has some noteworthy strengths. First, it included HZ cases diagnosed identified based on ICD-9 codes or, in some of the cases, by adding objective laboratory tools. 

Second, the semi-national database was supplied by CHS, the largest health supply service in the southern district, where our study was conducted. The large number of HZ cases documented in the post-varicella vaccination era showed a clear trend between cases and vaccination. By collecting data regarding patients managed as outpatients only, as well as those who had to be hospitalized due to HZ and its complications, we derived a significant amount of information and deciphered its clinically important consequences.

A third advantage of our work that differentiates it from former HZ and varicella vaccination-related studies is that our study population has unique characteristics, comprising two ethnic groups. The originality of this study is highlighted by some specific characteristics, such as a known vaccination adherence rate, a nearly single healthcare provider in the community, and a single regional tertiary hospital that provides some geographic isolation.

Finally, additional characteristics addressed are the patients’ ages and genders, which contribute another layer of distinction between the pre- and post-vaccination eras and enable a link between several facts to form a possible trend.

### 4.2. Limitations

The limitations of our study in part occurred because it involves ecological research. Thus, the relationships among variables at the population level may not be the same as at the individual level. In addition, its retrospective nature limits our ability to address clinical questions that necessitate a direct association, partly since, although HZ cases were diagnosed in part by valid laboratory objective measures, no distinction was made regarding whether or not the putative cases of zoster were due to vOka (the live varicella vaccine) or wild-type VZV. Another unexplained finding left to be studied in future works is the relatively large number of patients in the 0- to 1-year-old age group without a documented preceding case of varicella, perhaps due to congenital varicella syndrome or vertical transfer during labor, which needs to be further studied. That the study included data on the pediatric population alone leaves a concern regarding future HZ major morbidity and mortality in other populations, as no major disabilities or deaths were documented as part of the complications in the study population.

The difference between the results of our study and those conducted in different countries also raises several questions that remain unanswered in this current work. For example, possible explanations for more extensive HZ reports in our study may include either guidance shortage before vaccination, a nearly free public health system that lead to more significant numbers of clinic/hospital visits in Israel, or other unknown factors to be further studied in the future.

## 5. Conclusions

In conclusion, we demonstrated a significant increase in HZ cases in southern Israel following the initiation of mandatory varicella vaccination in 2008 but without a change in the HZ complication rate. Additionally, we offered several indicators that may explain this increase and perhaps connect it to the implantation of vaccinations against VZV mandated by the government during this time period. 

Further prospective studies are warranted to address this issue and validate our findings to prove our hypothesis. Collecting additional data, such as zoster sine herpete cases, and determining whether the putative cases of zoster were due to vOka or wild-type VZV have the potential to render our conclusion an association rather than a trend.

## Figures and Tables

**Table 1:** Study population data, taken from a big data managing program called “Bina” encompassing information on all children aged 0–18 years old who live in Israel’s southern district and are insured by the CHS.

Sector + Gender	Age Classification	0–1	1–2	2–6	6–12	12–18
**Jewish Females**	Incidence 2000–2007	24.98	86.32	33.09	32.33	20.77
	Incidence 2008–2021	22.34	77.61	62.95	104.90	207.29
	Incidence Diff—Cases per 100k	−2.64	−8.71	29.86	72.57	186.52
	Incidence diff—%	0.89	0.90	1.90	3.24	9.98
**Arab Females**	Incidence 2000–2007	58.72	180.07	43.15	79.13	65.68
	Incidence 2008–2021	173.02	414.98	307.62	266.75	507.59
	Incidence Diff—Cases per 100k	114.30	234.91	264.47	187.62	441.91
	Incidence diff—%	2.95	2.30	7.13	3.37	7.73
**Jewish Males**	Incidence 2000–2007	19.74	0	27	18.31	23.4
	Incidence 2008–2021	24.41	72.01	67.25	90.46	189.04
	Incidence Diff—Cases per 100k	4.67	72.01	40.25	72.15	165.64
	Incidence diff—%	1.24	-	2.49	4.94	8.08
**Arab Males**	Incidence 2000–2007	0	114.42	69.5	53.66	32.97
	Incidence 2008–2021	151.61	423.19	313.13	286.78	535.33
	Incidence Diff—Cases per 100k	151.61	308.77	243.63	233.12	502.36
	Incidence diff—%	-	3.70	4.51	5.34	16.24

**Table 2:** Complications incident rates.

Diagnosis Period	Total Cases of Complications	Population	Complications Incidence (×100,000 Population)
2000–2007	5	117,522	4.25
2008–2021	16	132,538	12.07

## Data Availability

The data are not publicly available due to ethical restrictions. We have full control of all primary data and agree to allow the journal to review the data if requested. Further enquiries can be directed to the corresponding author.
